# Consanguinity Mapping of Congenital Heart Disease in a South Indian Population

**DOI:** 10.1371/journal.pone.0010286

**Published:** 2010-04-21

**Authors:** Tracy L. McGregor, Amit Misri, Jackie Bartlett, Guilherme Orabona, Richard D. Friedman, David Sexton, Sunita Maheshwari, Thomas M. Morgan

**Affiliations:** 1 Department of Pediatrics, Vanderbilt University School of Medicine and the Monroe Carell Jr. Children's Hospital at Vanderbilt, Nashville, Tennessee, United States of America; 2 Narayana Hrudayalaya Institute of Cardiac Sciences, Bangalore, India; 3 Center for Human Genetics Research, Department of Molecular Physiology and Biophysics, Vanderbilt University Medical Center, Nashville, Tennessee, United States of America; Leiden University Medical Center, Netherlands

## Abstract

**Background:**

Parental consanguinity is a risk factor for congenital heart disease (CHD) worldwide, suggesting that a recessive inheritance model may contribute substantially to CHD. In Bangalore, India, uncle-niece and first cousin marriages are common, presenting the opportunity for an international study involving consanguinity mapping of structural CHD. We sought to explore the recessive model of CHD by conducting a genome-wide linkage analysis utilizing high-density oligonucleotide microarrays and enrolling 83 CHD probands born to unaffected consanguineous parents.

**Methodology/Principal Findings:**

In this linkage scan involving single nucleotide polymorphism (SNP) markers, the threshold for genome-wide statistical significance was set at the standard log-of-odds (LOD) score threshold of 3.3, corresponding to 1995∶1 odds in favor of linkage. We identified a maximal single-point LOD score of 3.76 (5754∶1 odds) implicating linkage of CHD with the major allele (G) of rs1055061 on chromosome 14 in the *HOMEZ* gene, a ubiquitously expressed transcription factor containing leucine zipper as well as zinc finger motifs. Re-sequencing of *HOMEZ* exons did not reveal causative mutations in Indian probands. In addition, genotyping of the linked allele (G) in 325 U.S. CHD cases revealed neither genotypic nor allele frequency differences in varied CHD cases compared to 605 non-CHD controls.

**Conclusions/Significance:**

Despite the statistical power of the consanguinity mapping approach, no single gene of major effect could be convincingly identified in a clinically heterogeneous sample of Indian CHD cases born to consanguineous parents. However, we are unable to exclude the possibility that noncoding regions of *HOMEZ* may harbor recessive mutations leading to CHD in the Indian population. Further research involving large multinational cohorts of patients with specific subtypes of CHD is needed to attempt replication of the observed linkage peak on chromosome 14. In addition, we anticipate that a targeted re-sequencing approach may complement linkage analysis in future studies of recessive mutation detection in CHD.

## Introduction

Congenital heart disease (CHD) affects approximately one percent of newborn infants [1], [2], and accounts for one third of deaths due to congenital malformations [3]. Worldwide, parental consanguinity confers a two to three fold increase in risk for a broad range of CHD phenotypes, as reported in Saudi Arabia [4], [5], Lebanon [6], and South India [7], [8]. This implies that recessive inheritance contributes to CHD occurrence. Consanguinity is already known to contribute to recessive Mendelian syndromes in which CHD is a feature. For example, Ellis van Creveld syndrome, due to homozygous mutations in *EVC* or *EVC2* [9], [10], presents with short stature, postaxial polydactyly, hidrotic ectodermal dysplasia, and a broad range of cardiac defects, most frequently septal defects or common atrium. The underlying genetic etiology was identified in genetically isolated Amish families [11]. Although syndromic CHD due to autosomal recessive mutations has been conclusively demonstrated, few genetic linkage investigations of nonsyndromic CHD have been performed in relation to parental consanguinity. One notable example identified *PDA1* as a candidate locus for patent ductus arteriosus in an Iranian population [12].

The consanguinity mapping approach has not yet been widely applied to nonsyndromic CHD, in part because parental consanguinity is uncommon in places where research efforts have historically been most intense. However, interest is growing in the statistical power of this approach for genetic investigations of other complex phenotypes, such as autism, as demonstrated by the Homozygosity Mapping Collaborative for Autism [13]–[17]. The main advantage of consanguinity mapping is that a relatively small number of pedigrees is required; as few as three families in which a single affected child is born to first cousin parents can result in 1000∶1 odds in favor of linkage, assuming no genetic heterogeneity and tight linkage of a disease gene with fully informative DNA markers [18]. This approach is also known as “autozygosity mapping,” because it assumes the identical-by-descent co-transmission of a particular ancestral mutation by both parents to an affected child. As such, this approach is only suitable for consanguineous pedigrees.

CHD presents major public health challenges in India, due to the large annual number of births in the Indian population and the limited resources for specialized care of children with CHD [19]. Consanguineous marriages are traditional in South India, with uncle-niece and cousin marriages being most common [20]. Studying a recessive model of CHD in this population is therefore feasible. Thus, we ascertained CHD patients born to consanguineous parents in India and referred to a center in Bangalore, representing a broad range of structural heart malformations. We hypothesized that consanguinity mapping would identify autosomal recessive mutations responsible for one or more CHD phenotypes in this population. In order to accomplish this aim, we performed a genome-wide linkage screen using dense oligonucleotide arrays in CHD probands and their consanguineous parents.

## Results

**Figure 1:**
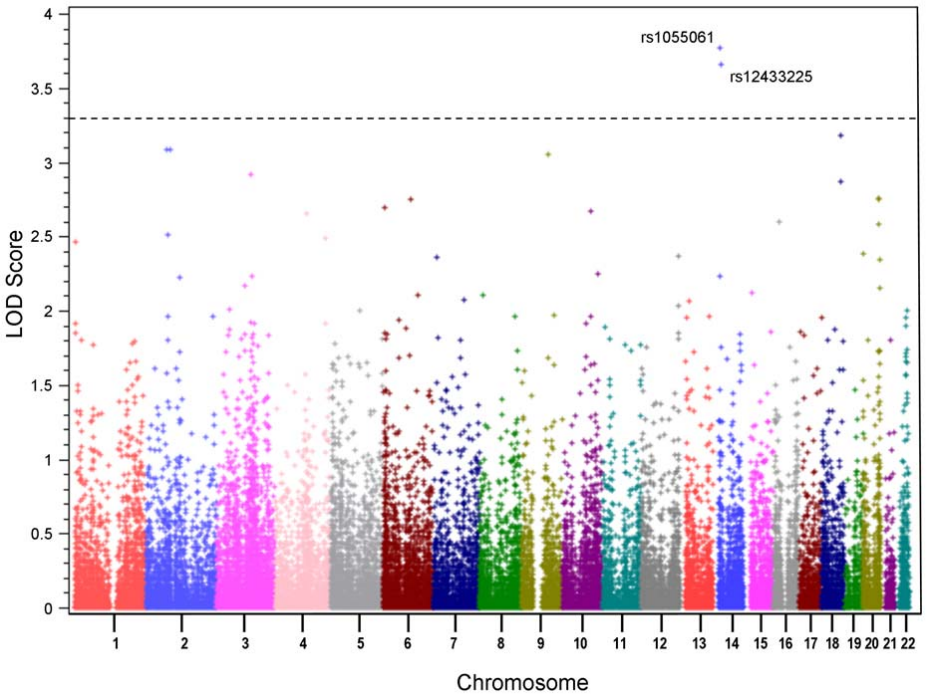
Manhattan plot of results of linkage analysis. Dashed line indicates genome wide significant LOD score of 3.3. Significant SNPs, rs1055061 and rs12433225 are represented.

The phenotypic distributions of the cohorts are described in Table 1. Initial screen with homozygosity mapping in the discovery cohort did not reveal any regions in which all probands were autozygous, as expected with a heterogeneous population. Two-point linkage analysis resulted in two SNPs with LOD scores above the standard threshold of 3.3 (Figure 1), indicating at least 1995∶1 odds in favor of linkage [21]. The first SNP, rs1055061, lies in the gene *HOMEZ* on chromosome 14 at 22,814,772. The LOD score was 3.76 (5754∶1 odds), and the optimal theta value was 0.05. The genotype frequencies of the probands were GG = 0.83, GA = 0.13 and AA = 0.04. The second SNP, rs12433225, yielded a LOD score of 3.65 (4467∶1 odds) and maps to chromosome 14 at 25,596,730, more than 250 kb away from any known gene in either direction. As a supplemental analysis, we also conducted a TDT test of association using WASP, and obtained a significant p-value of 0.0495 for rs1055061 with the major allele (G) overtransmitted and a non-significant p-value of 0.8185 for rs12433225.

**Table 1:** Phenotypic distributions for affected patients in both the discovery and replication cohorts.

Phenotype	Discovery Cohort	Replication Cohort
Conotruncal Lesions	28 (33.7%)	70 (21.6%)
Left Sided Obstructive Lesions	0 (0%)	2 (0.6%)
Right Sided Obstructive Lesions	5 (6%)	20 (6.2%)
Septal Defects	37 (44.6%)	123 (38%)
Single Ventricle Lesions	1 (1.2%)	83 (25.6%)
Valvular Defects	3 (3.6%)	18 (5.6%)
Complex Defects*	9 (10.8%)	8 (2.5%)
Total	83	325

*Complex defects refer to those lesions that had elements of multiple classes or did not conform to the preceding classifications.

Each of the linked SNPs was examined in the context of autozygosity of the probands. SNP rs1055061 resided in a region of autozygosity in 13 patients, while SNP rs12433225 localized to a region of autozygosity in 14 patients. Analysis of 39 other SNPs randomly selected from the Affymetrix platform resulted in a mean of 6.6 probands (SE = 0.4; SD = 2.6) with autozygosity surrounding the region of interest. Additionally, the minimum region of autozygosity shared by the 13 patients surrounding rs1055061 is Chr14:21,071,230–24,393,433, and the minimum region of autozygosity shared by the 14 patients surrounding rs12433225 is Chr14:24,952,386–25,774,000.

We re-sequenced the coding regions of *HOMEZ* in 54 probands. On sequence analysis of the two coding exons, four previously known SNPs [22] were identified in addition to rs1055061, namely rs1057119, rs10131813, rs67447855, and rs71800228. In addition, three patients exhibited changes not previously described. One proband had a heterozygous alteration in the 5′ UTR region (c.-116G>A), another displayed a Gly10Arg substitution in exon 1, and a third patient had a heterozygous deletion of T in the 3′ UTR (c.+193delT).

The population in our linkage analysis was comprised of children of consanguineous Indian parents. To determine if the *HOMEZ* SNP rs1055061 was also associated with CHD in an American population, we genotyped cases and controls from this population to assess allele frequency differences using an ABI TaqMan assay. Frequencies were 282 GG, 42 GA, and 1 AA in 325 patients with CHD and 548 GG, 57 GA and no AA in 605 control patients. The difference in the frequency of the GG genotype in cases versus controls was not statistically significant by Chi-squared analysis (P = 0.074). Secondary analysis of an allele frequency difference between cases and controls was not statistically significant by Chi squared analysis (P = 0.062).

## Discussion

We have presented statistically significant linkage evidence for a potential CHD susceptibility locus on chromosome 14 in a South Indian population selected for consanguinity. However, the linkage finding was not robust in a genetic association follow-up study of CHD in the general United States population. Utilizing the extensive genotyping information in oligonucleotide arrays, linkage analysis revealed two markers with LOD scores above 3.3. Because approximately 2.8 Mb separates these markers, a distance far in excess of typical linkage disequilibrium values in the Indian population [23], we conclude that these two SNPs are not independently linked to a separate single causative mutation.

Of the two markers, only rs1055061 is intragenic, causing a non-synonymous amino acid substitution. This SNP lies within *HOMEZ*, a vertebrate specific homeobox gene with an interesting structural organization. This transcription factor harbors three atypical homeodomains, two leucine zipper-like motifs, and an acidic domain. The protein is ubiquitously expressed in human tissues, and has been further studied in a murine developmental model [24]. Specific activity and pathway of this protein has not yet been elucidated. Disease associated mutations in human *HOMEZ* have not been described [25] and SNPs in this gene have not been associated with any human phenotypes [22].

The rs1055061 SNP identified by linkage induces an amino acid substitution from the polar, positively charged arginine to the polar, but neutral glutamine. The allele with the higher population frequency was overtransmitted in the TDT analysis, whereas one might expect a rare recessive allele to confer susceptibility to an uncommon phenotype such as CHD. Linkage analysis resulted in a theta value of 0.05, indicating that a causative mutation most likely lies within 50 kb of this SNP. Additionally, an increased number of probands exhibited a region of autozygosity surrounding each of the linked SNPs, supporting our hypothesis of autosomal recessive inheritance due to parental consanguinity. We do acknowledge that the LOD score of 3.76 indicates a 1∶5754 odds of this finding being due to chance alone, but argue that the convergence of multiple methods of inquiry support rs1055061 as a true finding.

We thus proceeded with sequence analysis of the coding regions of *HOMEZ* in a subset of the Indian probands with the hypothesis that a deleterious mutation in linkage disequilibrium with rs1055061 would be discovered. We detected previously reported SNPs and three novel sequence variations. None of the novel variations were found to be homozygous, as would be expected for causative mutations in offspring of a consanguineous relationship. Thus, we were unable to identify any causative homozygous mutations in the coding regions of *HOMEZ*.

We also studied the rs1055061 SNP in an American cohort of patients affected with various types of CHD and compared them to population controls. No significant difference was detected in the genotype or allele distribution between cases and controls, suggesting that our linkage finding in South Indians does not extend to genetic association in an American population of mixed European ancestry.

Although this study did not reveal definitive evidence of causal mutations leading to autosomal recessive CHD, a number of future directions and refinements are suggested. A limitation in the application of consanguinity mapping to CHD is that the fraction of this heterogeneous group of diseases attributable to recessive inheritance and the approximate number of loci involved is not known. Ascertaining consanguineous families with multiple affected children (suggesting recessive inheritance) would theoretically reduce genetic heterogeneity and increase power for linkage. If only multiplex pedigrees are enrolled, the proportion of probands with sporadic or autosomal dominant forms of CHD will be minimized.

We did not restrict our analysis to specific CHD phenotypes because it is impossible to select *a priori* which forms of CHD share a common genetic etiology. This may have resulted in diffusion of the linkage signal because of both clinical and genetic heterogeneity. Enriching the study population for predefined, specific phenotypic classes would likely decrease genetic heterogeneity in the study population.

Recent work in massively parallel whole exomic [26] as well as whole genomic [27], [28] re-sequencing suggests complimentary or alternative approaches to supplement current methods in consanguinity mapping. As technologies advance, identification of linked regions followed by targeted capture and deep resequencing of these regions with the aim of detecting locus-specific recessive mutations in multiple affected individuals may further enhance the power of consanguinity mapping. The increased power of this approach may allow for the suggested modifications of utilizing more informative and complex families while concentrating on specific forms of CHD in order to decrease genetic heterogeneity.

## Materials and Methods

### Ethics Statement

Written informed consent was provided by the parents of all children assenting to participate in the study, using consent documents approved by the ethics board of Narayana Hrudayalaya Heart Hospital. Dr. Sunita Maheshwari, Principal Investigator at Narayana Hrudayalaya Heart Hospital, and Dr. Amit Misri, co-investigator, obtained consent from participating families. Genotyping was performed on de-identified samples without knowledge of any personal identifiers under IRB approval from Yale University, Washington University in St. Louis, and Vanderbilt University.

### Population

We enrolled children affected with congenital heart disease and born to consanguineous parents in order to identify candidate regions for CHD by linkage analysis. The families were ascertained in a cardiology clinic at Narayana Hrudayalaya in Bangalore, India, from 07/2004 to 07/2007. The inclusion criteria were the presence of structural congenital heart disease and parental consanguinity. Exclusion criteria were isolated conduction system defect, cardiomyopathy, or extracardiac syndromic features. The patients had an echocardiogram performed in the clinic and interpreted by pediatric cardiologists. In all, 83 probands were recruited from 81 families. Probands were not known to be related to each other with the exception of one family that enrolled three affected siblings. Both parents were recruited in 71/81 families, and one parent in 10/81 families. The phenotypic distribution of the probands' cardiac lesions is described in Table 1, and the median age was 5.3 years (interquartile range 1.3–11.0 years). The parental relationships were uncle-niece for 42/81 families and first cousins for 39/81 families.

### Replication Cohort

A replication cohort of 325 probands affected with congenital heart disease was ascertained at Vanderbilt University Medical Center via therapeutic clinical trials of oral [29] or intravenous citrulline [30] for prevention of pulmonary hypertension. The CHD cases were compared with 605 population-based controls without CHD, the characteristics of whom were described previously [31]. Neither probands nor controls were known to be the product of a consanguineous relationship and nearly all were White, with a small number of cases (n = 39) and controls (n = 15) being of mixed Hispanic ancestry. Because the minor allele frequency of rs1055061 varies by population (the A allele has highest frequency in Africans and lowest in Whites), we restricted our analysis to White and Hispanics to account for this known population variation.

Genomic DNA for probands 1–26; 39–50 and relatives was isolated from blood spots on Whatman filter paper using GenSolve blood spot DNA recovery kit according to the manufacturer's protocol (Whatman Inc., Florham Park, NJ). DNA from probands 27–38 was isolated from peripheral blood samples using Gentra PureGene Blood Kit (Qiagen, Valencia, CA). For probands 51–83 and relatives, saliva was collected with Oragene kits, and DNA was extracted per manufacturer's protocol (DNA Genotek Inc., Kanata, Ontario, Canada). We performed genome wide scans utilizing Affymetrix SNP 5 microarrays for probands 1–30 and relatives with genotyping reactions performed at Precision Biomarker Resources (Evanston, IL). Probands 39–83 and relatives were genotyped on Affymetrix SNP 6.0 microarrays, and genotyping reactions were performed according to the manufacturer's instructions (Affymetrix, Santa Clara, CA). Genotypes were generated using the BRLMM-P for Affymetrix SNP 5 arrays and the Birdseed V-2 algorithm for Affymetrix SNP 6.0 arrays as released in Genotyping console v3.0.1 (Affymetrix, Santa Clara, CA). The SNP 5 arrays had an average QC call rate of 94.5% with SD of 4.8%. The SNP 6 arrays had an average QC call rate of 91.2% with SD of 5.6%. The genotype data was exported as .chp files and further analyzed with Merlin,v1.1.2 [32]. Genotypes on the sex chromosomes were excluded from the analysis, as these would not fit the hypothesized recessive model. Genotypes for SNPs present on both platforms were selected for analysis (n = 427,829). Quality control filters were run sequentially using the Whole-genome Association Study Pipeline (WASP) [33]. SNPs with genotype efficiency <95% (n = 268,653), a minor allele frequency <10% (n = 68,415), or Hardy Weinberg equilibrium with p<0.0001 (n = 867) were excluded. Because of the consanguineous nature of our population, we performed linkage analysis assuming a recessive mode of inheritance. Assuming a disease frequency of 0.01 and penetrance of 0.5, two-point analysis was conducted on the 89,894 SNPs that passed filtering criteria using Merlin. Since the Transmission Disequilibrium Test (TDT) is robust for linkage in the presence of inbred populations [34], TDT was performed for the SNPs with LOD >3.3 utilizing trios informative for each locus. All SNP citations and positions are given in reference to dbSNP build 129 [22] and the UCSC human genome browser [35] with the March 2006 assembly.

Assessment of regions of autozygosity were conducted in Partek software, V6.4 Copyright 2009 (Partek Inc., St. Louis, MO) with the algorithms for loss of heterozygosity (LOH). Unpaired analysis parameters included max probability 0.99, genomic decay 0, genotype error 0.02, and default frequency 0.3. All genotype calls from the probands were used to generate array specific baselines. Random markers were selected from the Affymetrix SNP5 & SNP6 platforms using a random number generator. Each result from the LOH analysis overlapping the genomic location of interest was visually inspected to determine true autozygosity.

We carried out a mutation screening of *HOMEZ* with bidirectional sequencing in a subset of Indian probands (n = 54). Four primer sets covering the coding exons and their flanking regions were designed with Primer3 (http://frodo.wi.mit.edu/cgi-bin/primer3/primer3_www.cgi). Fragment amplification was performed under standard PCR conditions. In brief, 50 ng of DNA was used in the reaction; PCR program started with 94°C for 5 min, 35 cycles of 94°C, 57°C and 72°C, a final extension cycle of 72°C for 6 minutes and stored at 4°C. PCR products were purified using Exo-SAP (Fermentas) and sequenced with the ABI 3730xl DNA Analyzer at the DNA Sequencing Facility, Vanderbilt University.

For the replication cohort, a proprietary TaqMan genotyping assay for rs1055061 was obtained from Applied Biosystems (Assay ID: C___2484687_10). After lyophilizing 10 ng genomic DNA per sample, the genotyping assay and TaqMan Universal PCR Master Mix (Applied Biosystems 4371355) were then prepared and run as per manufacturer's protocol. Applied Biosystems SDS® 2.3 was used to analyze the results of the genotyping assay. Genotypes were assigned without knowledge of the affected status of the samples.
